# Novel DNA methylation signatures of tobacco smoking with trans-ethnic effects

**DOI:** 10.1186/s13148-021-01018-4

**Published:** 2021-02-16

**Authors:** C. Christiansen, J. E. Castillo-Fernandez, A. Domingo-Relloso, W. Zhao, J. S. El-Sayed Moustafa, P.-C. Tsai, J. Maddock, K. Haack, S. A. Cole, S. L. R. Kardia, M. Molokhia, M. Suderman, C. Power, C. Relton, A. Wong, D. Kuh, A. Goodman, K. S. Small, J. A. Smith, M. Tellez-Plaza, A. Navas-Acien, G. B. Ploubidis, R. Hardy, J. T. Bell

**Affiliations:** 1 Department of Twin Research and Genetic Epidemiology, King’s College London, London, UK; 2 Department of Environmental Health Sciences, Columbia University Mailman School of Public Health, New York, USA; 3 Department of Chronic Diseases Epidemiology, National Center for Epidemiology, Carlos III Health Institute, Madrid, Spain; 4 Department of Statistics and Operative Research, University of Valencia, Valencia, Spain; 5 Department of Epidemiology, School of Public Health, University of Michigan, Ann Arbor, USA; 6 MRC Unit for Lifelong Health and Ageing, Institute of Cardiovascular Science, University College London, London, UK; 7 Population Health Program, Texas Biomedical Research Institute, San Antonio, USA; 8 School of Population Health and Environmental Sciences, King’s College London, London, UK; 9 MRC Integrative Epidemiology Unit, University of Bristol, Bristol, UK; 10 Population, Policy and Practice Research and Teaching Department, UCL Great Ormond Street Institute of Child Health, London, UK; 11 Centre for Longitudinal Studies, UCL Social Research Institute, University College London, London, UK; 12 Department of Biomedical Sciences, Chang Gung University, Taoyuan, Taiwan; 13 Genomic Medicine Research Core Laboratory, Chang Gung Memorial Hospital, Linkou, Taiwan

**Keywords:** Smoking, Epigenetics, DNA methylation, Environment, Lifestyle, SLAMF7

## Abstract

**Background:**

Smoking remains one of the leading preventable causes of death. Smoking leaves a strong signature on the blood methylome as shown in multiple studies using the Infinium HumanMethylation450 BeadChip. Here, we explore novel blood methylation smoking signals on the Illumina MethylationEPIC BeadChip (EPIC) array, which also targets novel CpG-sites in enhancers.

**Method:**

A smoking-methylation meta-analysis was carried out using EPIC DNA methylation profiles in 1407 blood samples from four UK population-based cohorts, including the MRC National Survey for Health and Development (NSHD) or 1946 British birth cohort, the National Child Development Study (NCDS) or 1958 birth cohort, the 1970 British Cohort Study (BCS70), and the TwinsUK cohort (TwinsUK). The overall discovery sample included 269 current, 497 former, and 643 never smokers. Replication was pursued in 3425 trans-ethnic samples, including 2325 American Indian individuals participating in the Strong Heart Study (SHS) in 1989–1991 and 1100 African-American participants in the Genetic Epidemiology Network of Arteriopathy Study (GENOA).

**Results:**

Altogether 952 CpG-sites in 500 genes were differentially methylated between smokers and never smokers after Bonferroni correction. There were 526 novel smoking-associated CpG-sites only profiled by the EPIC array, of which 486 (92%) replicated in a meta-analysis of the American Indian and African-American samples. Novel CpG sites mapped both to genes containing previously identified smoking-methylation signals and to 80 novel genes not previously linked to smoking, with the strongest novel signal in *SLAMF7*. Comparison of former versus never smokers identified that 37 of these sites were persistently differentially methylated after cessation, where 16 represented novel signals only profiled by the EPIC array. We observed a depletion of smoking-associated signals in CpG islands and an enrichment in enhancer regions, consistent with previous results.

**Conclusion:**

This study identified novel smoking-associated signals as possible biomarkers of exposure to smoking and may help improve our understanding of smoking-related disease risk.

## Background

Tobacco smoking is one of the leading preventable causes of death [46] and a leading risk factor for disease burden [16]. Smoking damages the airways and induces lung disease, such as chronic obstructive pulmonary disease (COPD), lung cancer and increased risk of multiple long-term conditions including heart disease and stroke [43]. Further wide-ranging health risks associated with smoking relate to several other cancers (including oral, gastro-intestinal, urinary tract and reproductive cancers), bone health, gum disease, macular degeneration, type 2 diabetes, rheumatoid arthritis and altered immune function [15, 16].

Smoking exposure has been robustly associated with changes in DNA methylation. Many smoking-associated CpG-sites, or differentially methylated positions (DMPs), have been identified and replicated to date [18, 25]. The majority of recent studies linking blood DNA methylation levels with smoking exposure have used the Infinium HumanMethylation450 BeadChip (450k array) to generate genome-wide DNA methylation profiles. In the largest smoking-methylation study of adults to date [25], Illumina 450k methylation profiles for 15,907 blood samples from 16 cohorts were used to identify 2623 smoking-DMPs in or near to 1405 genes. Many of these DMPs were already linked to smoking by previous studies [7, 49]. The most consistently associated CpG-sites across independent studies are located in or near the *AHRR, RARA, PRSS23, F2RL3, GPR15 and GNG12* genes and in chromosomal regions *2q37.1* and *6p21.33*. These epigenetic signals have also been used to develop DNA methylation-based biomarkers of smoking status such as EpiSmokEr, currently the most robust smoking classifier [5].

Recently human genome-wide DNA methylation studies have started to explore the additional genome coverage afforded by the new Infinium MethylationEPIC BeadChip (EPIC array), which assays 850,000 CpG-sites at nearly double the coverage of the Illumina 450k array. Recent findings based on DNA methylation profiles in lung tissue [37] and saliva [2] identified smoking-associated methylation signals profiled only by the EPIC array, which suggests that novel smoking EPIC methylation signals may also be identified in blood. Here, we assessed the association between smoking and DNA methylation levels profiled using the Illumina EPIC array in 1407 whole blood samples from four UK population cohorts and pursued replication in independent cohorts. Our analyses compared DNA methylation levels between current and never smokers in a cross-sectional analysis to identify novel smoking-associated differentially methylated positions (smoking-DMPs). The observed signals were subsequently compared between former and never smokers to assess whether these alterations persist after smoking cessation. The results have potential to improve existing biomarkers of smoking that could be used to infer smoking exposure where it is not known. Furthermore, the findings may identify novel genes which may explain specific disease risk mechanisms in smokers.

## Methods

### Discovery phase participants

The participants in the discovery phase included 1407 individuals from four UK population cohorts—TwinsUK and three birth cohorts, including the 1970 British Cohort Study (BCS70), the National Child Development Study (NCDS) or 1958 British birth cohort, and the MRC National Survey of Health and Development (NSHD) or 1946 British birth cohort (Additional file 1: Note). The sample included 235 individuals from BCS70, 529 individuals from NCDS, 236 individuals from the NSHD and 407 individuals from TwinsUK (Table 1). The majority of participants included for DNA methylation profiling were not selected for a specific phenotype distribution or environmental exposure, although sample selection strategies included minimizing exposure or phenotype data missingness across samples (see Additional file 1: Note). An exception was a subset of 294 participants in the NCDS cohort selected for extremes of child and adulthood adversity [6, 41] (see Additional file 1: Note). Smoking information was obtained from questionnaire data collected at the time of DNA methylation profiling. All study participants provided informed consent, and ethical approval was granted by local research ethics committees (see Additional file 1: Note). The overall discovery sample included 269 current, 495 former and 643 never smokers from the UK population (see Additional file 1: Note).

**Table 1:** Sample characteristics

Cohort^a^	Sample size	Smoking status^b^			Sex		Age (years)	BMI	Ethnicity^c^
		S (n)	F (n)	NS (n)	F (%)	M (%)			
Discovery stage									
TwinsUK	407	23	237	147	100	0	64 ± 8	26.8 ± 5.0	White British
NSHD	236	23	75	138	60	40	63 ± 0.5	27.9 ± 4.7	White British
NCDS1	235	53	118	64	53	47	45	25.6 ± 4.3	White British
NCDS2	294	125	87	82	51	49	45	25.8 ± 4.3	White British
BCS70	235	45	126	64	57	43	46	26.6 ± 5.0	White British
Replication stage									
SHS	2325	893	648	748	59	41	55 ± 6	29.6 ± 3.0	American Indian
GENOA	1100	179	255	666	71	29	56 ± 10	31 ± 6.6	African-American

^a^Cohort abbreviations: TwinsUK: https://twinsuk.ac.uk/, NSHD: MRC National Survey of Health and Development, NCDS: National Child Development Study (NCDS1 = selected to minimize data missingness, but not selected for specific exposures and trait outcomes. NCDS2 = selected for extremes of child and adulthood adversity), BCS70: 1970 British Cohort Study, SHS: Strong Heart Study. GENOA: Genetic Epidemiology Network of Arteriopathy

^b^Smoking status, age and BMI are obtained at the date of blood draw for DNA methylation profiling. Smoking status (S = smoker, F = former smoker, NS = never smoker) is determined through questionnaires

^c^Participants from the UK population cohorts are predominantly “White British.” NCDS2 includes one individual who did not identify as “White British” (“Mixed”). BCS70 includes three individuals who did not identify as “White British” (one “White Other”, one “White and Asian” and one “Other Ethnic Group”)

### DNA methylation profiles

DNA samples extracted from whole blood were profiled using the Illumina MethylationEPIC BeadChip (Illumina EPIC) array, which targets over 850,000 CpG-sites including more than 90% of the probes on the 450k and additional CpG-sites predominantly in enhancers [36]. DNA methylation levels were determined using methylation beta-values, defined as the ratio of the methylated bead signal to the sum of the unmethylated bead signal plus the methylated bead signal plus 100 [10]. Methylation beta-values range between 0 at unmethylated CpG-sites and 1 at fully methylated CpG-sites. DNA methylation quality control measures included multiple checks and normalizations. Altogether, 72,471 probes were identified as cross-reactive or polymorphic and excluded from the analysis. Cross-reactive probes were defined as those mapping to multiple locations of the in silico bisulfite converted human genome allowing for two mismatches. Probes were also excluded if they targeted polymorphic CpG sites with minor allele frequency (MAF) > 5% in the UK10K haplotype reference panel. Probes with greater than 5% missingness were also excluded from the analysis, spanning 1348 probes. DNA methylation levels were then normalized using ENmix [48]. The number of CpG-sites included in downstream analyses was 710,658.

### Peripheral blood cell proportions

A significant difference in blood cell-type proportions has previously been reported between smokers and never smokers [32]. This finding, along with the observation that DNA methylation profiles also vary by blood cell type, highlights the need to take into account blood cell proportion differences in the analyses. Blood cell-type proportions were estimated for monocytes, granulocytes, immune cells (Natural Killer (NK) cells, CD8 and CD4) and plasmablasts using the approach proposed by Houseman et al. [22]. Correlations between estimated blood cell types were investigated to inform a set of covariates for inclusion in epigenetic linear models of association. As a result, blood cell subtype covariates used in downstream analyses included monocytes, granulocytes, NK cells and CD8-naive cells.

### Epigenetic association analyses

Epigenome-wide association scans (EWAS) were carried out within each cohort dataset, followed by meta-analyses. DNA methylation values at each CpG-site were normalized to *N*(0, 1) prior to fitting linear models. EWAS focused on the comparison of DNA methylation profiles between smokers and never smokers at 710,658 CpG-sites genome-wide. Linear models of association, run separately for each cohort dataset, compared DNA methylation values as the response variable to smoking status as a predictor. For the three birth cohorts, linear models were fitted (lm function in R) where normalized methylation level at each CpG-site was the response variable and predictors included smoking status, sex, blood cell-type proportion, BMI and methylation chip and position of the sample on the chip. EWAS were run separately in the two NCDS cohort subsets, due to different sample selection strategies and proportion of smokers. In the TwinsUK sample linear mixed-effects models were fitted using lme4 [3] and lmerTest [27] in R, using the same fixed-effects covariates as for the birth cohort models excluding sex because all twins were female, but including age as well as random effects variables for family and zygosity.

Following individual cohort sample EWAS, a fixed-effects inverse variance weighted meta-analysis was applied to combine results across cohorts. Meta-analysis, performed using GWAMA [34], was carried out across the 5 datasets of 1407 subjects in total. To minimize effects attributed to heterogeneity across cohorts we only considered meta-analysis results that did not exhibit strong evidence for heterogeneity (Qp < 10% and an *I*^2^ > 50%, [21]). Multiple-testing adjustment of the meta-analysis results was performed using both the Benjamini and Hochberg false discovery rate (FDR) threshold (FDR = 1%) and a Bonferroni-corrected threshold (*P* value = 6.25 × 10^−8^ ≈ 0.05/800,000), with both sets of results reported. Methylation effect sizes were calculated using the same linear models, but without normalizing DNA methylation levels to *N*(0, 1) prior to data analysis. Evidence for genomic inflation was assessed using *λ*, which is the ratio of the median of the empirically observed distribution of the test statistic to the expected median, thus quantifying the extent of the bulk inflation and the excess false-positive rate. Results from the largest smoking-methylation study to date, Joehanes et al. [25] (at FDR < 0.05), were used to assess whether smoking-DMPs are novel.

Once smoking differentially methylated positions (smoking-DMPs) were identified from the meta-analysis, follow-up analysis included a comparison of DNA methylation levels between former and never smokers at the 952 smoking-DMPs. In these follow-up analyses a Bonferroni-corrected threshold was applied for multiple testing (*P* value = 5.25 × 10^−5^ ≈ 0.05/952).

### Replication in trans-ethnic samples

Many of the previously established smoke exposure effects on DNA methylation have been robustly replicated across different ethnicities. With this in mind and to assess whether the signals that we detected are conserved across different ethnic populations, we pursued replication in two samples of non-European ancestry. Replication of the novel smoking-DMPs was carried out firstly in American Indian participants from the Strong Heart Study [28] and secondly in African-American participants from the Genetic Epidemiology Network of Arteriopathy study [8] (see Additional file 1: Note).

The first replication sample consisted of 2325 American Indians aged 45–74 (893 smokers and 684 never smokers). DNA methylation was measured using the EPIC array in whole blood samples collected in 1989–1991. Pre-processing was conducted according to Illumina’s recommendations, and snoob and Regression on Correlated Probes (RCP) normalizations were applied [13, 35]. Batch effects by sample plate, sample row and DNA isolation time were corrected using comBat (sva R package). Peripheral blood cell proportions were estimated as in the other cohorts. Replication was pursued at 525 of novel smoking-DMPs profiled by the EPIC array only that also passed quality control assessment in the replication dataset. The same covariates and modeling approach as for the birth year cohorts was used in the replication sample.

The second replication sample consisted of 1100 African-Americans with a mean age of 56 (179 smokers and 666 never smokers). A total of 1106 samples at GENOA Phase I and 304 samples at GENOA Phase II were assessed using the Illumina HumanMethylationEPIC BeadChip. Raw IDAT files were imported using Minfi R package [1]. The shinyMethyl R package [13] was used to visualize the raw intensity data and identify sex mismatches and outliers were removed. Individual probes with detection *P* value < 10e−16 were considered to be detected successfully [29], and samples and probes with detection rate < 10% were removed. Samples with incomplete bisulfite conversion identified using the QCinfo() function in the ENmix R package were removed [48]. Sample identity was checked using the 59 SNP probes implemented in the EPIC chip and mismatched samples removed. Next, Noob was used for individual background and dye-bias normalization [14]. Since two types of probes are present on the EPIC BeadChip (Infinium I and Infinium II), we used the RCP method to adjust for probe-type bias [35]. After exclusions, a total of 857,121 probes in 1100 samples at Phase I and 294 samples at Phase II were available for analysis. Peripheral blood cell proportions were estimated as in the other cohorts. Replication was pursued at 526 of novel smoking-DMPs profiled by the EPIC array only. Linear mixed-effects models were fitted using the same methodology as for the TwinsUK cohort (lme4 and lmerTest), and the same fixed-effects covariates as for the birth cohort models but including age as well as a random effect variable for sibship. Meta-analysis across the two replication samples was carried out using the same methodology as for the main analysis. A Bonferroni-corrected threshold was applied (*P* value = 9.5 × 10^−5^ ≈ 0.05/525), and only results showing the same direction of association as in the discovery sample were considered.

### SLAMF7 gene expression analysis

Gene expression analysis for *SLAMF7* was carried out in a sample from the TwinsUK cohort with available whole blood gene expression data. RNA-seq data generation and pre-processing have been previously described in detail [17]. In summary, STAR software v2.4.0.1 [12] was used to align reads to the hg19 reference genome. Samples with fewer than 10 million aligned reads were excluded. Following this process, there were 383 whole blood samples remaining including 227 never smokers, 30 current smokers and 126 former smokers, where 162 individuals overlapped with the TwinsUK sample used in the main analysis. Gene counts were transformed into trimmed mean of M-values (TMM)-adjusted counts per million (CPMs) and inverse-normalized prior to all downstream analyses.

A mixed effect linear model was fitted (lme4) with smoking as the predictor and gene expression as the response. Covariates included fixed effects: insert-size median, mean GC content and random effects: primer index, date of sequencing, zygosity, family and RNA extraction batch. A *P* value was determined using lmerTest with a significance threshold of 0.05.

### Genomic annotation and pathway analysis

Genomic annotation of smoking-DMPs was carried out initially using the EPIC Illumina manifest, for the purpose of identifying novel CpG-sites that are specific to the EPIC-array, for allocating CpG-sites to genes, and relative to CpG-density, including CpG island (CGI), CGI-shore, CGI-shelf, and open sea. Further genomic annotations took into account data from the ENCODE project [20]. We explored if smoking-DMPs mapped within ChromHMM [11] categories to assess enrichment or depletion in smoking-DMPs relative to different functional genomic domains, including insulators, enhancers and specific transcription factors binding sites. In the enrichment analysis, we considered all smoking-DMP probes mapping to a specific annotation category compared to the total number of probes tested that mapped to that category. For each genome annotation category, the results show the log fold change for smoking-DMP probes compared to total probes tested, and the significance of the difference is based on a Fisher’s exact test. Pathway analysis was carried out for genes annotated to smoking-DMPs using Ingenuity Pathway Analysis (IPA; QIAGEN Inc. https://www.qiagenbioinformatics.com/products/ingenuitypathway-analysis).

### Prediction of smoker status

We tested several models for classifying smoking status based on subsets of the newly identified smoking-DMPs. The sensitivity and specificity of each classification was assessed using receiver operative curve (ROC), implemented using the pROC package in R [38]. The analyses were carried out in the combined dataset of 1113 subjects who were not selected for phenotypic extremes, excluding the NCDS subset of 294 individuals. Training datasets were created taking 60% of the combined dataset at random. A test dataset was created with the remaining 40%. Altogether, 20 random samples were taken creating 20 random training and test set combinations.

For each of the training datasets, a generalized linear model was fitted based on the predictors, including un-adjusted DNA methylation levels at the candidate CpG-site(s) and covariates (age, sex, blood cell-type proportions, BMI, methylation chip and position of the sample on the chip) using the R glm function. The test dataset was then loaded into the derived model with outcomes predicted using the R predict function. The average AUC was determined for each methylation value combination.

Methylation combinations for distinguishing between current smokers and never smokers included cg05575921 (*AHRR*) and cg00045592 (*SLAMF7*) on their own, and then in combination.

Methylation combinations for distinguishing any smoke exposure (that is, either current or former smoker) and never smokers were explored in three models. The first model included the 5 ex-smoking DMPs with the largest effect size in the 450k array (cg21566642, cg05575921, cg01940273, cg25189904, cg12803068), the second model included the 5 ex-smoking DMPs with largest effect size sites from the EPIC array (cg14391737, cg21566642, cg05575921, cg25189904, cg05533761), and the third model included the 5 ex-smoking DMPs with the largest effect size overall (cg14391737, cg21566642, cg05575921, cg25189904, cg05533761).

## Results

DNA methylation profiles and smoking were explored in 1407 total whole blood samples from 4 UK population cohorts in the discovery stage (269 current smokers, 495 ex-smokers and 643 never smokers), and in 3425 whole blood samples from American Indians and African-Americans in the replication stage (Table 1). The primary analyses focused on identification of smoking differentially methylated positions (smoking-DMPs) between current smokers and never smokers, and follow-ups explored smoking-DMPs genomic distribution, pathway analysis, stability upon smoking cessation, and predictive value.

### Epigenome-wide association analysis: current versus never smokers

**Fig. 1:**
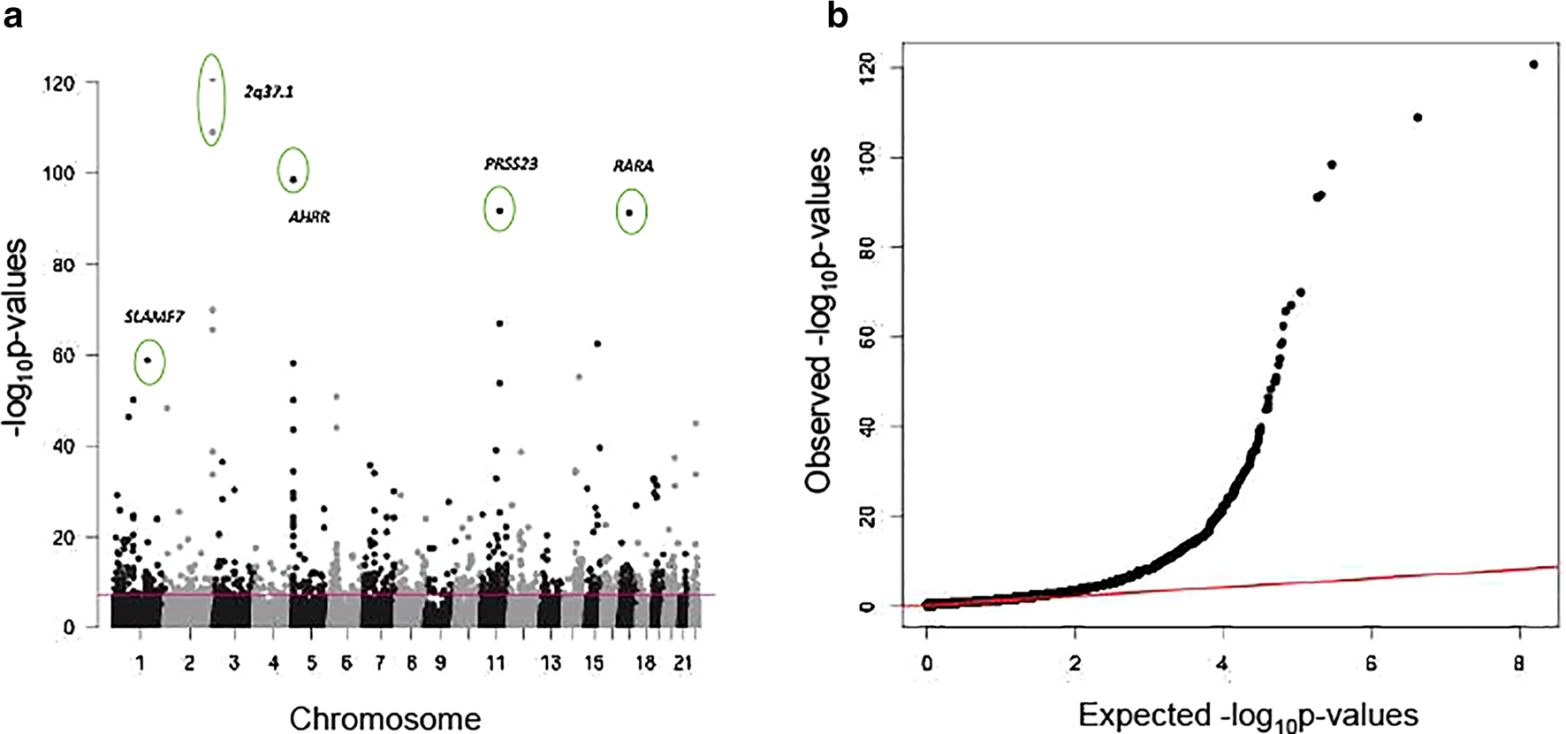
Methylation association results in current versus never smokers. **a** Manhattan plot of genome-wide results for methylation association with smoking. Smoking-DMPs are indicated above the Bonferroni-adjusted threshold (red line). **b** Quantile–quantile (QQ) plot for CpG-site association in current versus never smoker

Meta-analysis comparing DNA methylation profiles in current smokers (*N* = 269) and never smokers (*N* = 643) identified 952 CpG sites or smoking-DMPs in 500 genes that were statistically differentially methylated at a Bonferroni-adjusted threshold (*P* value = 6.25 × 10^−8^). At a more relaxed threshold (FDR 1%), there were 3348 CpG sites in 1632 genes (Additional file 2: Table S1). There was evidence for genomic inflation (see Methods) with an overall *λ* of 1.28 (Fig. 1a), which is consistent with other meta-analyses of smoke exposure [25]. Smoking-DMPs are spread across the genome, consistent with previous observations (Fig. 1b).

Of the 952 smoking-associated CpG-sites, 422 have previously been identified [25] and are represented on both the 450 k array and the EPIC array (Additional file 2: Table S2). The strongest association was observed at cg21566642 in the *2q37.1* region (*P* value = 1.6 × 10^−121^), which has been observed previously in multiple studies. Furthermore, cg05575921 in *AHRR*, which is the most frequently observed association in previous studies, was the most significantly associated CpG site in our study that was also annotated to a gene (*P* = 2.9 × 10^−99^). The effect size at this site, based on unadjusted DNA methylation beta values, was also the largest where current smokers exhibited on average 25% lower DNA methylation values compared to never smokers, which is also consistent with previous studies [25, 49].

**Fig. 2:**
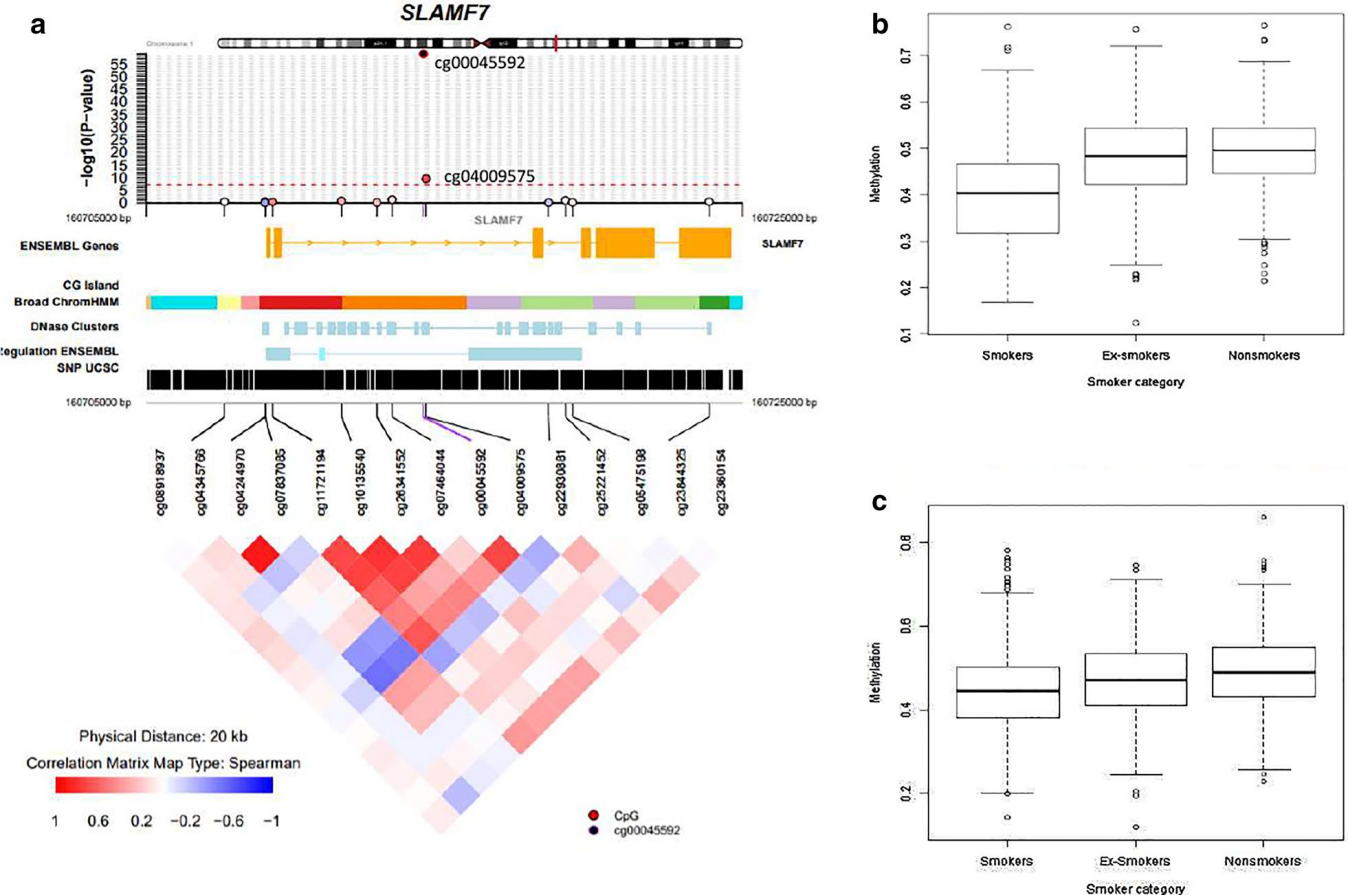
Novel smoking-associated DNA methylation signals in *SLAMF7.* **a** coMET plot [33] describing the genomic region of epigenome-wide association between smoking and SLAMF7 methylation (top panel) showing the two smoking-DMPs cg00045592 and cg04009575, along with functional annotation of the region (middle panel) where broad ChromHMM regions are displayed using UCSC genome browser color schemes, and pattern of co-methylation at the 12 CpG sites in the EPIC array annotated to *SLAMF7* (bottom panel). **b** Boxplot showing a comparison of DNA methylation levels at cg00045592 between smokers, former smokers and never smokers in the combined TwinsUK, NCDS, NSHD, BCS70 data set. **c** Boxplot showing a comparison of DNA methylation levels at cg04009575 between smokers, former smokers and never smokers in the Strong Heart Study

Of the 952 significant CpG-sites, 526 smoking-DMPs represent novel CpG sites only profiled by the EPIC array (Additional file 2: Table S2). The 526 smoking-DMPs were annotated to 277 genes, of which 80 genes have not previously been linked to differential methylation with smoking. The most significant novel CpG-site, cg00045592, was annotated to gene *SLAMF7,* (*P* value = 1.39 × 10^−59^), which has not been previously linked to methylation changes with smoking (Fig. 2). The effect size, or the unadjusted mean methylation in non-smokers compared to smokers, was 10% change in mean methylation, representing one of the larger effect sizes (13th largest). Furthermore, a second DMP at this gene, site cg04009575, was also identified as a smoking-DMP (*P* value = 3.02 × 10^−10^). In addition to novel DMPs mapping to the 80 novel genes, many other novel DMPs mapped to genes linked to smoking by CpG sites measured by the Illumina 450k array. Examples include cg1431737 in *PRSS23* (*P* value = 6.5 × 10^–92^) and cg17739917 in *RARA* (*P* value = 1.97 × 10^–92^). Altogether, 68% of novel DMPs are upstream of or within genes, with 20% in new genes and 48% in genes where methylation changes have previously been associated with smoking.

The majority of smoking-DMP effects were hypomethylated in current smokers (74% of Bonferroni-adjusted sites, and 90% of the 100 sites with the lowest associated *P* values). The average effect sizes, measured as the difference in mean unadjusted methylation levels between never smokers and current smokers, were broadly similar between the hypomethylated (average effect size of 3.3% for hypomethylated sites) and hypermethylated (average effect size of 2.2% for hypermethylated) signals. However, the largest effect sizes detected overall (up to 25% mean difference) were observed at hypomethylated sites.

### Replication of novel smoking signals

Novel smoking-DMPs that were profiled only by the EPIC array were evaluated for replication. Many smoking signals have previously been replicated across ancestries, and therefore we tested whether our results were robust across different ancestries. Replication was pursued in two independent samples, the first included 2325 American Indian participants from the Strong Heart Study [28], and the second included 1100 African-American participants in the Genetic Epidemiology Network of Arteriopathy (GENOA) study. A recent study in the SHS sample by Domingo-Relloso et al. [9] analyzed EPIC array profiles of whole blood samples for smoke exposure in connection with cadmium levels in urine and also identified novel smoking-DMPs. In addition to the distinct ancestry, this cohort also has a higher proportion of smokers, and furthermore one of the leading causes of death for American Indians is CVD, for which smoking is a risk factor. Of the 526 novel sites, 389 (74%) replicated in the SHS sample alone at a Bonferroni-corrected threshold (*P* value = 9.5 × 10^–5^) with the same direction of association. This included cg00045592, annotated to gene *SLAMF7*, which replicated with a *P* value of 1.45 × 10^–23^. At nominal significance (*P* value = 0.05), there were 500 (95%) novel sites with the same direction of association effect. The GENOA sample also has distinct ancestry, and African-Americans have the highest incidence of hypertension, for which smoking is a risk factor. Recent analysis of the dataset validated smoking-DMPs identified in saliva [2]. Of the 526 novel sites, 418 (79%) replicated at a Bonferroni-corrected threshold (*P* value = 9.5 × 10^–5^) with the same direction of association in the GENOA sample alone. This also included cg00045592, annotated to gene *SLAMF7*, which replicated with a *P* value of 7.57 × 10^–31^. At nominal significance (*P* value = 0.05), there were 508 (97%) novel sites with the same direction of association effect.

In a meta-analysis of the two replication cohorts in altogether 3425 individuals, 486 (92%) of the 526 novel smoking-DMPs replicated at a Bonferroni-corrected threshold (*P* value = 9.5 × 10^–5^) with the same direction of association. This also included cg00045592, annotated to gene *SLAMF7*, which replicated with a *P* value of 3.54 × 10^–51^. At nominal significance (*P* value = 0.05), there were 522 (99%) novel sites with the same direction of association effect (Additional file 2: Table S4).

### Genome annotation analysis

**Fig. 3:**
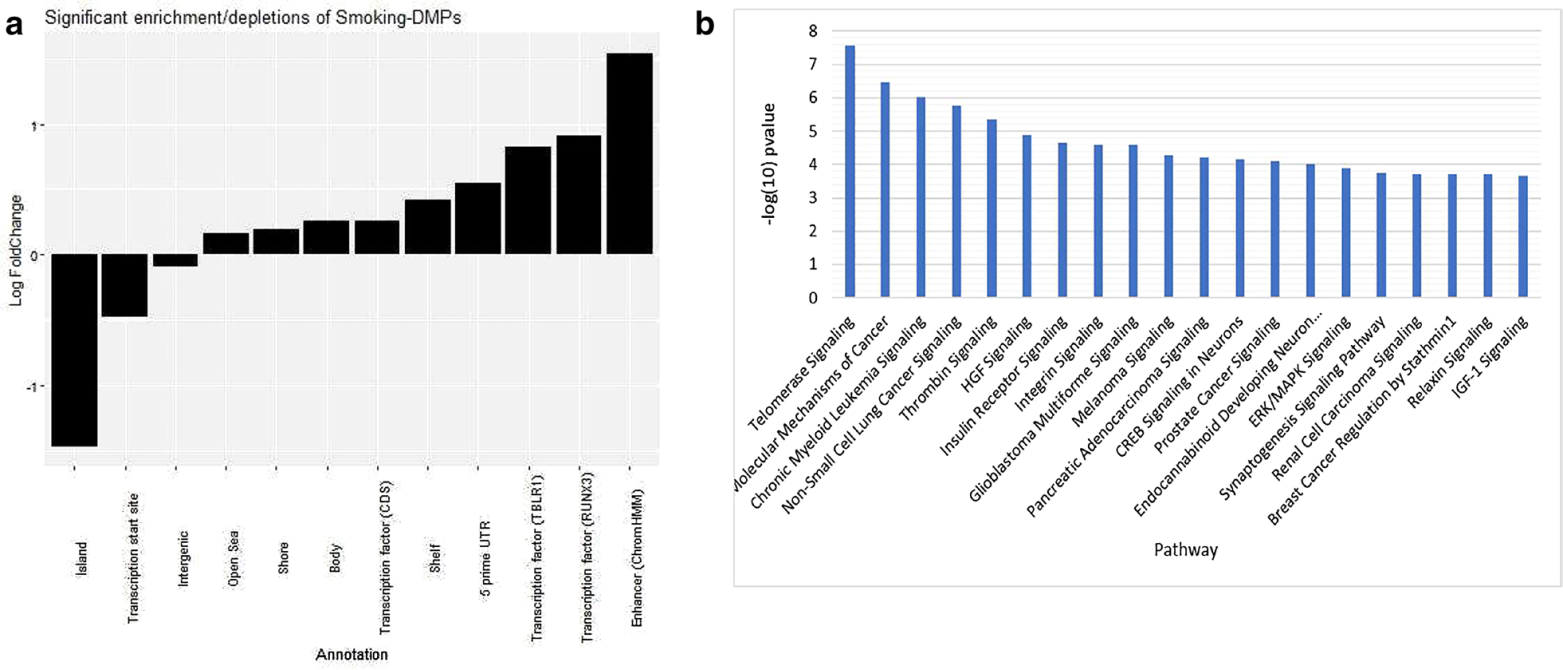
Smoking-DMPs annotation and pathway analysis. **a** Log fold changes relating to the proportion of probes annotated to particular genomic locations and the proportion of smoking-DMPs. Only annotation categories with statistically significant results based on Fisher’s exact test are shown **b**. IPA canonical pathway analysis, 20 lowest *P* values shown

We assessed the genome distribution for all 952 smoking-DMPs identified in our study, relative to all probes assayed by the EPIC array. We explored enrichment and depletion of smoking-DMPs across different genomic categories. The enrichment analysis considered 18 genomic annotation categories (Fig. 3a). The strongest effect was a clear enrichment of smoking-DMPs in enhancer regions as predicted by ChromHMM (36% of smoking-DMPs relative to 12% of probes tested), consistent with previous studies [25] and with the hypothesis that smoking exposure impacts regulatory genomic features. Furthermore, also consistent with previous work, there was an enrichment of smoking-DMPs in gene bodies (50% of smoking-DMPs relative to 42% of probes tested). Finally, there was a depletion of smoking-DMPs in CpG islands (7% relative to 18% of tested probes), which is consistent with previous observations that CpG islands are less dynamic in response to exposure [50].

### Pathway analysis

The 952 smoking-DMPs were annotated to 500 genes. We explored evidence for enrichment of these genes within different biological processes, focusing on canonical pathways using IPA (see Methods). Altogether, the 500 genes identified enrichment for 101 molecular pathways (*P* value = 0.01, Additional file 2: Table S5). Among the 20 most-enriched pathways, 8 (40%) relate to cancer, with the remainder relating to cell signaling and growth pathways, neuronal health, cardiovascular health and insulin receptor activity (Fig. 3b).

### SLAMF7 gene expression follow-up analysis

To assess potential functional impacts of the novel smoking-DMPs identified in *SLAMF7*, we explored *SLAMF7* gene expression levels in smokers and never smokers. The analysis was carried out in 383 blood samples from the TwinsUK cohort with available blood RNAseq levels [17]. A nominally significant difference in *SLAMF7* gene expression (*P* = 0.02) was detected where current smokers had reduced levels of expression of *SLAMF7*.

### Epigenome-wide association analysis: former versus never smokers

To assess how smoking-DMPs behave after smoking cessation, we carried out a cross-sectional analysis comparing DNA methylation levels in 497 former and 643 never smokers at the 952 smoking DMPs. If DNA methylation levels at smoking DMPs persist after smoking cessation, we would expect significant differences in DNA methylation at all 952 signals, as we did in the comparison of current and never smokers. However, we observed that at the majority of smoking-DMPs there was no significant difference between former and never smokers, suggesting reversal of smoking-associated DNA methylation levels upon smoking cessation. Altogether, there were 37 differentially methylated sites at a Bonferroni-adjusted threshold (*P* value = 1.5 × 10^–5^), at which our results were consistent with persistent effects after smoking cessation (41 signals at FDR 1%, Additional file 2: Table S3). Of the 37 Bonferroni signals, 16 represented novel signals only profiled on the EPIC array, 20 replicated previously identified persistent smoking-methylation signals observed by Joehanes et al. [25] present on both arrays, and one signal profiled on both arrays was novel. The 16 novel EPIC-specific signals annotated to 11 genes, and the most significant signal in a novel gene was in *SLAMF7* (cg00045592, *P* value = 8.8 × 10^–7^), where former smokers have on average around 2.4% lower unadjusted methylation levels than never smokers. The majority (92%) of the 37 signals are hypomethylated in former smokers, and the effect sizes were smaller than observed between the smokers and never smokers. On average, the mean effect size across all 37 signals was a hypomethylation of 2% in former smokers, and the largest effect size obtained was 6% (cg14391737).

### Methylation-based detection of smoking status

We assessed the performance of the peak smoking-DMPs as classifiers of smoking status. The DNA methylation level at our peak novel smoking-DMP, cg00045592 (*SLAMF7*), is a moderately good classifier for distinguishing between current smokers and never smokers with an AUC of 0.87. The overall peak smoking-DMP and widely replicated cg05575921 (*AHRR*) is a much stronger classifier between smokers and never smokers with an AUC of 0.95. Adding *SLAMF7* to *AHRR* marginally improves the AUC to 0.96. We next explored the predictive value of different DMPs to distinguish current or former exposure to smoking (‘ever smoked’), by comparing current and former smokers to never smokers. Neither cg00045592 (*SLAMF7*) or cg05575921 (*AHRR*) are very good predictors of current or former smoke exposure compared to never smokers, with AUC values of 0.68 and 0.76, respectively. Using the 5 450 k-only array signals with the largest effect sizes from the comparison of former and never smokers (cg21566642, cg05575921, cg01940273, cg25189904, cg12803068) results in a slightly better classification of ever smoked exposure with an average AUC of 0.79 (Additional file 3: Figure S1a). The classifier is marginally improved by including novel EPIC-specific sites, where the 5 overall signals with the largest effect sizes from the comparison of former and never smokers (cg14391737, cg21566642, cg05575921, cg25189904, cg05533761) result in an average AUC of 0.80 (Additional file 3: Figure S1b).

## Discussion

The current study explored smoking status and DNA methylation levels profiled on the EPIC array in 1407 whole blood samples from individuals across 4 independent UK population-based cohorts. We identified 952 CpG smoking-DMPs between smokers and never smokers at Bonferroni adjustment. Of these, 422 replicated previous findings and 526 signals were novel, involving CpG sites profiled only by the EPIC array. Of the 526 novel signals, 486 replicated in the American Indian and African-American replication sample. The different ethnicity of the replication samples, along with the high proportion of smokers and their propensity for CVD in the American Indian population and prevalence of hypertension in the African-American population, indicates that our smoking-DMPs signals are robust and trans-ethnic. While many of the novel signals were in genes previously found to be associated with smoke exposure, 80 novel genes were also identified where the top novel signal was in the *SLAMF7* gene. The genes that harbored smoking-related methylation signals were enriched to fall in biological pathways related to cancer, cell signaling and growth pathways, neuronal health, cardiovascular health and insulin receptor activity. Although the majority of signals showed at least some evidence for reversal in DNA methylation levels with smoking cessation, we also identified smoking-DMPs which persisted after smoking cessation. Overall, this is consistent with previous studies and observations that upon smoking cessation risk of smoking-related diseases reverts to non-smoker levels over time [45], but for some outcomes risk is not fully reversible.

The most strongly associated novel smoking-DMP (cg00045592) is in a gene not previously linked to smoking, *SLAMF7,* which also contains a secondary smoking-DMP signal (cg04009575, ranked 489th of the 952 smoking-DMPs). Both sites are located in an enhancer and therefore likely to influence the expression of genes including *SLAMF7*. A follow-up analysis showed that smokers had significantly lower levels of expression of *SLAMF7*. Both signals replicated in both replication cohorts. DNA methylation at cg00045592 alone can distinguish current smokers and never smokers with an average AUC of 0.87. *SLAMF7 is* a protein coding gene and a member of the Signalling Lymphocyte Activation Molecule Family (SLAMF), a family of receptors with a role in both innate and adaptive immunity. *SLAMF7* is expressed on immune cells, recognizes and binds to itself, in turn leading to the downstream activation of natural killer cells [19]. Smokers have been shown to have suppressed NK activation [23], which in turn has been shown to lead to a reduction in NK tumor surveillance in smokers, and consequently an increased propensity for tumors [30]. *SLAMF7* has been linked to several diseases, for example, it is associated with systemic lupus erythematosus [26], multiple myeloma [24] and immunoregulatory interactions [31]. Methylation of *SLAMF7* has also been identified as a regulator in atherosclerosis [47]. *SLAMF7* has also been suggested as a possible therapeutic target for rheumatoid arthritis, a disease more common in smokers [44]. Our analysis showed that the differential methylation at cg0045592 persisted after smoking cessation, retaining on average at 2.4% lower DNA methylation levels compared to never smokers, which given its potential functional role may have implications for altered immune function in current and former smokers.

Genome annotation analysis showed a clear enrichment in smoking-DMPs in enhancer regions. Although this result has previously been observed on the 450k array in blood [25], a key strength of the EPIC array is the more detailed genome coverage of enhancers. This enrichment provides evidence that smoke exposure leads to methylation changes in regulatory regions, which in turn likely result in functional changes. This is consistent with previous studies which have shown gene expression changes as a result of smoke exposure [25, 42]. In addition, the rest of the annotation analysis showed results consistent with previous work [25, 50].

The results from the current study can be used to not only understand possible implications of smoke exposure on gene function, but also as potentially useful biomarkers of smoke exposure. Smoking is a confounder in many epidemiological studies given its wide-ranging impact on human health [43]. Not only is a molecular biomarker of smoking useful to confirm self-reported smoke exposure data, but it can be also valuable as a predictor of smoking in samples with missing smoking data, errors in self-reported status or in forensic settings. Previous work has developed predictors of current smoking based on 450k array smoking-DMPs, observing good predictive value (AUC of 0.95) of *AHRR* in particular as a smoking predictor. Our novel signals do not significantly improve smoking classification compared to using *AHRR* alone. However, a subset of smoking effects persists after cessation. Although we are unable to assess exactly how long these effects last for, they can be used as measures of current or previous smoke exposure. Therefore, our smoking-DMPs may be useful toward developing a classifier of present or past smoke exposure, that is, distinguishing current and former smokers from never smokers. In an attempt to address this, we observed an AUC of 0.80 for distinguishing present or past smoke exposure using the top 5 signals from both arrays. Including novel sites present only on the EPIC array affected prediction only marginally (0.79 vs 0.8). The results could also be used for further studies toward prediction of future smoking-related health outcomes.

Our findings may provide insights into molecular changes underlying tobacco smoke exposure risk effects in disease. Pathway analysis of the smoking-DMPs indicated that the genes annotated to smoking-DMPs are strongly linked to cancer, with 40% of the top 20 significant pathways relating to cancer. These included a number of different cancer types, not solely those relating to lung cancer, which concurs with epidemiological studies which show that smoking is a risk factor for several types of cancer. The methylome varies by tissue type, but we see a relationship between smoking-DMPs found in blood and those found in tissues directly relevant to some of these cancers, for example, in lung tissue. Specifically, there is an overlap between the smoking-DMPs identified here and those found by Ringh et al. [37] in lung tissue, where 32 of the 952 sites overlap particularly at sites annotated to *AHRR* and in genes previously found to be associated with cancer (*KCNMA1*, *CDH23, LRP5*) [[4, 39, 40]]. In addition to implications for cancer, the pathway analysis results also revealed strong links between smoking-DMPs and neuronal health. The novel gene findings are a starting point for analyzing whether and how DNA methylation alterations could lead to smoking-related disease.

There are some limitations to the current study. While the birth cohorts are not in themselves selective and should therefore lead to a broad sample representative of the population, not all individuals will continue to participate in cohort sweeps. In this study the majority of subjects were selected based on minimizing missing data across a range of variables, with low level of oversampling of specific subgroups. This approach could lead to some bias in sampling more engaged cohort participants. We observed genomic inflation in the smoking epigenome-wide analyses, which has also been observed in previous smoking-methylation meta-analysis reports [25]. Although this observation raises potential concerns about false positives, our results are in line with previous studies, replicating many previously reported signals, as well as 92% of novel signals in the trans-ethnic samples. We took a conservative approach by removing meta-analysis results that may be due to heterogeneity. This approach is likely to have removed some results that may well be true positives in some sample subsets. Whole blood samples are a mixture of different cell types, and we used methylation estimates of cell proportions to address this in the analysis. However, these are likely to be over generalizations and ideally analyses in specific cell subpopulations should be carried out. Another limitation is that DNA methylation was only studied in blood and not in other tissues (e.g., lung, adipose), which have previously been explored in the context of smoking on the 450k array. Other tissues such as lung tissue are likely to produce more insight into disease mechanisms, although with the exception of saliva, they lend themselves less well to biomarker detection due to the relative difficulty of sampling. Follow-up analyses, which our study did not explore, include a full gene-expression analysis to assess functional impacts at the novel smoking-DMPs, longitudinal analyses to characterize the stability of smoking-DMPs upon cessation over long timescales, and studies of these signals in samples of other ethnicities.

In conclusion, our study identified hundreds of novel smoking-methylation signals, including those annotated to genes not previously associated with smoke exposure. Some of the novel signals persist after cessation of smoking. The findings have potential to act as biomarkers of exposure to smoking and may improve our understanding of smoking-related disease risk.

## Supplementary Information

**Additional file 1: Note.**

**Additional file 2: Table S1.** Statistically significant CpGs in relation to current versus never smoking status at false discovery rate (FDR) < 0.01. **Table S2.** Statistically significant CpGs in relation to current versus never smoking status at a Bonferroni-adjusted threshold (*P* value = 6.25 × 10^–8^). **Table S3.** Statistically significant CpGs in relation to former versus never smoking status at false discovery rate (FDR) < 0.01. **Table S4.** Statistically significant CpGs in replication meta-analysis relation to current versus non-smoking status at a Bonferroni-adjusted threshold (*P* value = 9.5 × 10^−5^). **Table S5.** Pathway Analysis (IPA) results.

**Additional file 3: Figure S1.** Receiver operating characteristic (ROC) curves predicting current smokers and smoke exposure. **a** Top 5 CpG from 450 k only predicting smoke exposure and **b** Top 5 CpG from EPIC predicting smoke exposure.

## Data Availability

The majority of DNA methylation datasets in the current study, except those from the Strong Heart Study replication cohort, are available in the public domain. TwinsUK methylation data are uploaded on the ReShare UK Data Service, under Data collection id 853,526. NSHD methylation data access is through https://doi.org/10.5522/NSHD/S202. NCDS and BCS70 methylation data access is through https://doi.org/10.5255/UKDA-SN-5594-2. Access to further individual-level data including phenotype data can be applied for through each cohort data access committee. For information on access and how to apply, see https://twinsuk.ac.uk/resources-for-researchers/access-our-data/ (TwinsUK), http://www.nshd.mrc.ac.uk/data (NSHD), https://beta.ukdataservice.ac.uk/datacatalogue/series/series?id=2000032 (NCDS) and https://beta.ukdataservice.ac.uk/datacatalogue/series/series?id=200001 (BCS70). The Strong Heart Study DNA methylation data can be available to external investigators by following the procedures established by the Strong Heart Study Steering Committee in agreement with the study’s Tribal partners. These procedures are available at https://strongheartstudy.org/. Phenotype data for GENOA participants are available from the Database of Genotypes and Phenotypes (dbGaP): phs001401.v2.p1. Methylation data are from the Gene Expression Omnibus (GEO): GSE157131. Due to IRB restriction, mapping of the sample IDs between genotype data (dbGaP) and methylation data (GEO) cannot be provided publicly, but are available upon written request to JAS and SLRK.
