# Supplementary material for: Novel DNA methylation signatures of tobacco smoking with trans-ethnic effects
Source: Clin Epigenetics. 2021 Feb 16;13:36. doi: 10.1186/s13148-021-01018-4 (PMC7888173; doi:10.1186/s13148-021-01018-4)
Supplement: Supplementary file 1 — Additional file 1: Note. [file 13148_2021_1018_MOESM1_ESM.docx]

**Additional file 1: Note: Christiansen et al.**

**Study Cohorts and Participants**

**TwinsUK**

TwinsUK is the largest twin registry in the UK with over 14,000 identical and

non-identical same-sex twins (age 16-100). The registry was established in 1992 and is one of the most detailed studies of adult twins in the world. Extensive data are available for over a thousand phenotypes, environmental exposures, lifestyle and social factors. Biological samples and data are available in a large subset of cohort participants. The cohort and data collected have been previously described (Verdi et al, 2019).

All research participants have signed informed consent prior to taking part in any research activities. Ethical approval was granted by the National Research Ethics Service London-Westminster, the St Thomas' Research Ethics Committee (REC reference numbers: EC04/015 and 07/H0802/84).

*Cohort-specific Acknowledgments*

The TwinsUK study was funded by the Welcome Trust; European Community’s Seventh Framework Programme (FP7/2007-2013). The study also receives support from the National Institute for Health Research (NIHR)-funded BioResource, Clinical Research Facility and Biomedical Research Centre based at Guy's and St Thomas' NHS Foundation Trust in partnership with King's College London.

**The MRC National Survey of Health and Development (NSHD) or 1946 British birth cohort**

The NSHD is the oldest of the British birth cohort studies, with data on health and life

circumstances collected at multiple follow-ups from birth (>20) on 5,362 men and women born in England, Scotland and Wales in March 1946. Epigenetic profiles were obtained from blood DNA samples from 236 NSHD participants at age 60-64 when an intensive phenotype data collection was carried out, adding to a range and depth of existing prospective data across life. The cohort and the data collected has previously been described (Kuh et al, 2011; Wadsworth et al, 2005).

Ethical approval was granted by the Central Manchester Research Ethics Committee (07/H1008/168 and 07/H1008/245) and the Scotland A Research Ethics Committee (08/MRE00/12).

*Cohort-specific Acknowledgments*

The U.K. Medical Research Council provides core funding for the MRC National Survey of Health and Development (MC_UU_00019/1). We acknowledge study members for their lifelong participation and past and present members of the study teams, including members of the MRC Epidemiology unit in Cambridge, who helped to collect and process the data.

**The National Child Development Study (NCDS) or 1958 British birth cohort**

The NCDS is the second oldest of the British birth cohort studies. The initial sample of 17,415 individuals (8,411 females), consisting of all babies born in Great Britain in a single week in 1958, have had multiple follow-ups providing high quality prospective data on social, biological, physical, and psychological phenotypes at every sweep. Epigenetic profiles were obtained from DNA samples collected from 529 NCDS subjects at age 44-45, at the same time as intensive phenotyping during this biomedical follow-up. This cohort and the blood samples taken have been described previously (Power et al, 2005; Fuller et al, 2006).

Epigenetic profiles were generated for two NCDS samples. NCDS1, consisting of 234 subjects, was selected to minimise data missingness for a wide range of exposures of the lifecourse and phenotypes related to healthy ageing. NCDS1 subjects were not selected for particular exposures or outcomes, or for extremes of phenotype distribution. On the other hand, NCDS2, consisted of 294 subjects selected for extremes of child and adulthood adversity exposures. Briefly, the sample was selected according to a combinatorial exposure design for exposure either to low/high socio-economic position, childhood abuse or not, prenatal smoking or not, and bullying or not (Borghol et al, 2012; Suderman et al, 2014). DNA methylation profiles from NCDS1 and NCDS2 were profiled separately, but profiles were processed using the same procedure as described in the main manuscript (see Methods).

Ethical approval was granted by the London Central REC (14/LO/0097, 12/LO/2010 and 08/H0718/29) and by South East MREC (01/1/44). This covered consent for the collection of blood samples for health research. Biosamples for the sweep are held at University of Bristol and this has ethical approval as a tissue bank under application 09/H1010/12 from North-West Haydock NRES committee.

**The 1970 British Cohort Study (BCS70)**

BCS70 is a cohort study of all babies born in Great Britain in a single week in 1970. The

initial 16,569 individuals (7,975 females) have been surveyed at multiple (>8) sweeps. Epigenetic profiles were obtained from DNA samples collected from 235 BCS70 subjects at age 46, at the same time as intensive phenotyping during this first biomedical follow-up. This cohort has been described previously (Elliott et al, 2006).

Ethical approval was granted by the London Central REC (14/LO/1371, 11/LO/1560) and the Southampton & South West Hampshire REC 08/H0504/144 .

*NCDS and BCS70 Cohort-specific Acknowledgments*

We acknowledge the co-operation and participation of the individuals who voluntarily participate in the 1958 and 1970 birth cohort studies. We thank the Economic and Social Research Council for funding these cohorts through the Centre for Longitudinal Studies (CLS) at the UCL Institute of Education, London. We thank the Economic and Social Research Council for funding the Cross Cohort Research Programme (CCRP) (grant number: ES/M008584/1). Blood collection for the 1958 cohort was funded by the Medical Research Council (MRC) (grant G0000934 to the clinical examination and DNA banking of the 1958 cohort). We like to thank a large number of stakeholders from academic, policy-maker and funder communities and colleagues at CLS involved in data collection and management.

**The Strong Heart Study**

The Strong Heart Study (SHS) began in 1988. It is a study of cardiovascular disease and its risk factors among American Indian men and women. Three geographic areas are included: Arizona; Oklahoma; and North and South Dakota covering 12 tribes in total. In its initial stages, the SHS included three components. The first was a survey to determine cardiovascular disease mortality rates from 1984 to 1994 among tribal members aged 35 to 74 years who lived in the three study areas. The second was the clinical examination of 4,549 eligible tribal members. The third component was the morbidity and mortality surveillance of these 4,549 participants. The SHS has completed three clinical exams of the original cohort. The cohort has been described previously (Lee et al, 1990).

*Cohort-Specific Acknowledgements*

The Strong Heart Study has been funded in whole or in part with federal funds from the National Heart, Lung, and Blood Institute, National Institute of Health, Department of Health and Human Services, under contract numbers 75N92019D00027, 75N92019D00028, 75N92019D00029, & 75N92019D00030. The study was previously supported by research grants: R01HL109315, R01HL109301, R01HL109284, R01HL109282, and R01HL109319 and by cooperative agreements: U01HL41642, U01HL41652, U01HL41654, U01HL65520, and U01HL65521. The content is solely the responsibility of the authors and does not necessarily represent the official views of the National Institutes of Health.

**The Genetic Epidemiology Network of Arteriopathy**

The Genetic Epidemiology Network of Arteriopathy (GENOA) is a multi-phase, community-based, prospective study of sibships with two or more siblings diagnosed with primary hypertension before the age of 60. Participants self- identified as Black, additional siblings were invited to participate regardless of hypertension status, and recruitment took place in the Jackson, Mississippi area. A total of N=1,854 AA participants were recruited in Phase I (1995-2000) from 683 sibships, and in Phase II, N=1,482 returned (2000-2005). In each phase, demographics, medical history, clinical characteristics, lifestyle factors, and fasting blood samples were collected (Daniels, 2004).

*Cohort-Specific Acknowledgements*

Support for the Genetic Epidemiology Network of Arteriopathy (GENOA) was provided by the National Heart, Lung and Blood Institute (U01HL054457, RC1HL100185, R01HL119443, R01HL133221, and R01HL141292). We would also like to thank the families that participated in the GENOA study.

**References**

Borghol N, Suderman M, McArdle W, Racine A, Hallett M, Pembrey M, Hertzman C, Power C, Szyf M (2012) “Associations with early-life socio-economic position in adult DNA methylation” *International journal of epidemiology* **41**(1), 62–74.

Daniels P, Kardia S, Hanis C, Brown C, Hutchinson R, Boerwinkle E, Turner S and Genetic Epidemiology Network of Arteriopathy study (2004) “Familial Aggregation of Hypertension Treatment and Control in the Genetic Epidemiology Network of Arteriopathy (GENOA) Study”, *Am.J.Med*. **116**:676-681

Elliott J, Shepherd P (2006)“Cohort Profile: 1970 British Birth Cohort (BCS70)” *[Int J Epidemiol](https://academic.oup.com/ije/article/35/4/836/686544/Cohort-Profile-1970-British-Birth-Cohort-BCS70)***35**(4): 836-843

Fuller E, Power C, Shepherd P, Strachan D (2006)“Technical report on the National Child Development Study biomedical survey 2002–2004”. <https://cls.ucl.ac.uk/wp-content/uploads/2017/07/NCDS-biomed-technical-report.pdf>

Kuh D, Pierce M, Adams J, et al. (2011) “NSHD scientific and data collection team. Cohort profile: updating the cohort profile for the MRC National Survey of Health and Development: a new clinic-based data collection for ageing research” *Int J Epidemiol* **40**:e1–e9

Power C, Elliott J (2005) “Cohort profile: 1958 British birth cohort (National Child Development Study)” *Int J Epidemiol* **35**:34–41

Suderman M, Borghol N, Pappas J et al. (2014) “Childhood abuse is associated with methylation of multiple loci in adult DNA” *BMC Med Genomics* **7**(13)

Verdi S , Abbasian G, Bowyer R , Lachance G, Yarand D, Christofidou P, Mangino M, Menni C, Bell J.T, Falchi M, Small K, Williams F, Hammond C, Hart D, Spector T, Steves C, (2019) “TwinsUK: The adult twin registry update” *Twin Research and Human Genetics* **22**: 523–529

Wadsworth M, Kuh D, Richards M, Hardy R (2005) “Cohort profile: the 1946 National Birth Cohort (MRC National Survey of Health and Development)” *Int J Epidemiol***35**:49–54

Xu Z, Niu L, Li L,Taylor J (2016) “ENmix: a novel background correction method for Illumina HumanMethylation450 BeadChip” *Nucleic Acids Res*. **44**:e20
