# Supplementary figures and images for: Novel DNA methylation signatures of tobacco smoking with trans-ethnic effects
Source: Clin Epigenetics. 2021 Feb 16;13:36. doi: 10.1186/s13148-021-01018-4 (PMC7888173; doi:10.1186/s13148-021-01018-4)

1a

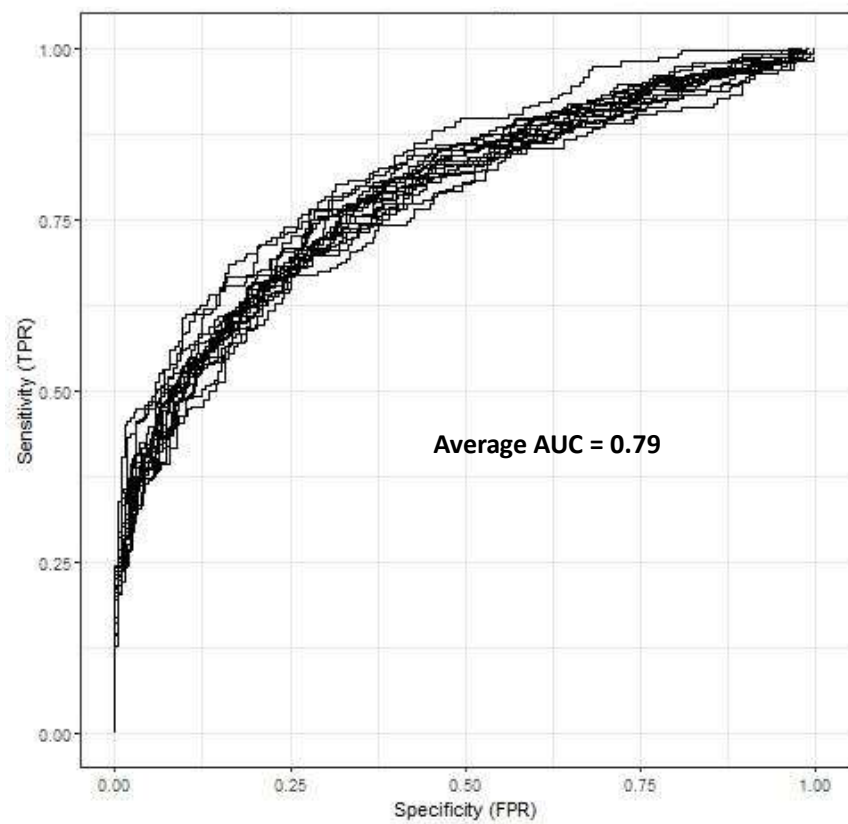

1b

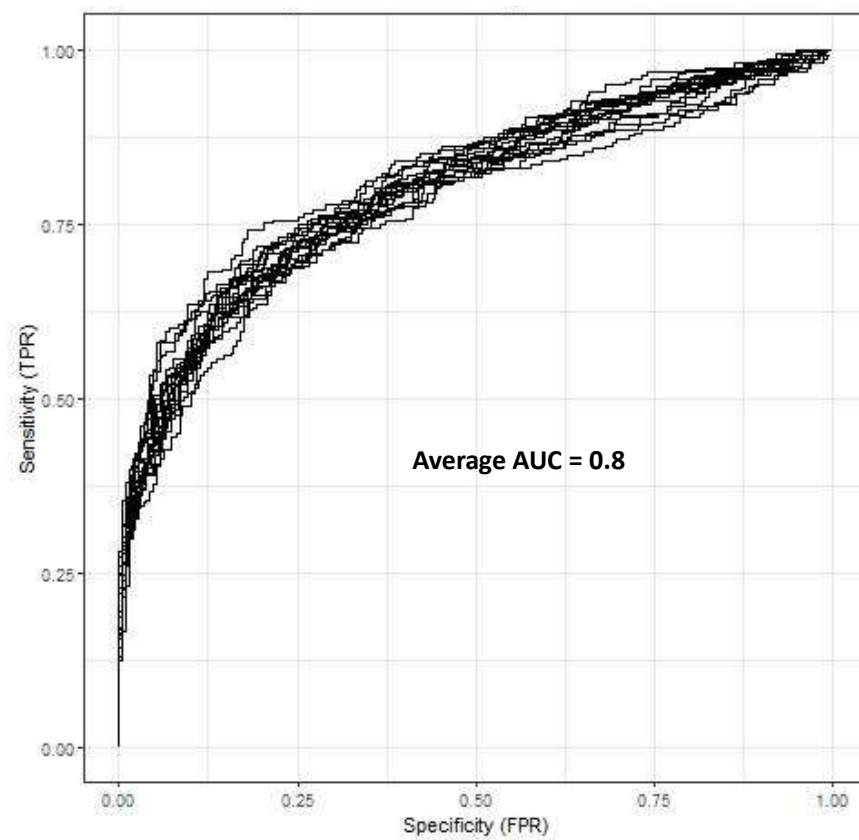

Supplement: Supplementary file 3 — Additional file 3: Figure S1. Receiver operating characteristic (ROC) curves predicting current smokers and smoke exposure. a Top 5 CpG from 450 k only predicting smoke exposure and b Top 5 CpG from EPIC predicting smoke exposure. [file 13148_2021_1018_MOESM3_ESM.pdf]
